# Welcome to the *World Allergy Organization Journal*!

**DOI:** 10.1097/wox.0b013e31816362c2

**Published:** 2008-01-15

**Authors:** Michael A Kaliner

**Affiliations:** 1 President, WAO, 2005-2007

## 

The world has gotten smaller, and continues to shrink! What goes on in Beijing or Cairo needs to be communicated to New York and Paris. To establish a rapid, efficient, and cost-effective communication system, the World Allergy Organization (WAO) has created a new unique on-line journal, the *World Allergy Organization Journal *or the *WAO Journal*. We envision this journal to be a clinically oriented communication, blending excellent reviews with clinically applied research. We have established an excellent international editorial board, contracted with Lippincott to publish the journal, and will fund the initial distribution, starting with volume 1, January 2008.

The WAO has made a major commitment to the success of this journal, which will be distributed to more than 25,000 allergists worldwide, ensuring that the *WAO Journal *will immediately reach more allergists than any other publication in the world. We can count on this immediate and extensive impact because our biweekly e-newsletter is a huge success: it has a vast global readership and is translated into 7 languages. We are actively soliciting both high-quality reviews and translational research articles for publication, and greatly look forward to receiving your submission for review.

After 18 years, the *Allergy and Clinical Immunology International *(ACII), the journal of the World Allergy Organization, has been discontinued. This journal was fun to read and was always interesting. However, it never achieved the readership it deserved or required for continuation, nor was it included in any of the citation indices. We thank those of our colleagues who supported ACII, particularly the past editors, Alain de Weck and Allen Kaplan, and the publisher Hogrefe and Huber. The WAO is giving a special recognition award to Rob Dimbleby of Hogrefe and Huber for his highly valued support.

Johannes Ring, the current editor of ACII, will transition to the editorship of the *WAO Journal*, joined by Lanny Rosenwasser as coeditor. They have assembled a group of world-renowned allergists as regional editors and an excellent editorial board.

Thus, the WAO recognizes the responsibility it has to facilitate excellence in allergy. We are committed to providing education through our lecture series (GLORIA, The Seminars and Conferences Lectureships, and the World Allergy Forum), Web site-related materials and case reports, our new webinars on immunology (cosponsored by the American Academy of Allergy Asthma and Immunology, and available soon on both our Web sites), a new series of on-line Continuing Medical Education programs based on clinical case histories in allergy, and the Emerging Societies Program (which, in partnership with the American College of Allergy, Asthma and Immunology, has continued to support educational activities in 2007 programs held in Thailand, China, and Latin America).

The WAO is now taking this next step to help the world of allergy communicate by sponsoring the new on-line journal. We promise you that it will maintain the highest standards with respect to both review articles and original research. We promise that the subscription cost of the journal will be inexpensive, and that the journal will arrive on-line and on time on your computer. Publication will be rapid and will focus on clinically relevant materials. The WAO journal will continue to be complemented by the biweekly e-newsletter, where important relevant articles will be summarized with commentary and news, and announcements from around the world will be communicated. Thus, you will be receiving continued on-line information from WAO, and the opportunity to communicate observations or information from wherever you work to the rest of world will achieve a new level of availability.

Yes, the world is shrinking, we are all working together at a new level of communication, and the WAO journal is an important cog in the wheel that will take us forward. We hope that you love and use this new journal. We all need to work together to ensure its success.

Michael A. Kaliner, MD

President, WAO, 2005-2007

